# Electrospun Structural Hybrids of Acyclovir-Polyacrylonitrile at Acyclovir for Modifying Drug Release

**DOI:** 10.3390/polym13244286

**Published:** 2021-12-07

**Authors:** He Lv, Shiri Guo, Gaoyi Zhang, Wanli He, Yonghui Wu, Deng-Guang Yu

**Affiliations:** 1 School of Materials and Chemistry, University of Shanghai for Science and Technology, Shanghai 200093, China; 201850158@st.usst.edu.cn (H.L.); 1935023610@st.usst.edu.cn (S.G.); 1935021211@st.usst.edu.cn (W.H.); 2 School of Optical-Electrical and Computer Engineering, University of Shanghai for Science and Technology, Shanghai 200093, China; 1935040829@st.usst.edu.cn; 3 The Department of Mechanical Engineering, Guangxi Technological College of Machinery and Electricity, Nanning 530007, China; 4 Shanghai Engineering Technology Research Center for High-Performance Medical Device Materials, Shanghai 200093, China

**Keywords:** coaxial electrospinning, structural hybrids, polymeric nanocomposites, modified release, medicated nanomaterials

## Abstract

In traditional pharmaceutics, drug–crystalline nanoparticles and drug–polymer composites are frequently explored for their ability to modify drug release profiles. In this study, a novel sort of hybrid with a coating of acyclovir crystalline nanoparticles on acyclovir-polyacrylonitrile composites was fabricated using modified, coaxial electrospinning processes. The developed acyclovir-polyacrylonitrile at the acyclovir nanohybrids was loaded with various amounts of acyclovir, which could be realized simply by adjusting the sheath fluid flow rates. Compared with the electrospun composite nanofibers from a single-fluid blending process, the nanohybrids showed advantages of modifying the acyclovir release profiles in the following aspects: (1) the initial release amount was more accurately and intentionally controlled; (2) the later sustained release was nearer to a zero-order kinetic process; and (3) the release amounts at different stages could be easily allocated by the sheath fluid flow rate. X-ray diffraction results verified that the acyclovir nanoparticles were in a crystalline state, and Fourier-transform infrared spectra verified that the drug acyclovir and the polymer polyacrylonitrile had a good compatibility. The protocols reported here could pave the way for developing new types of functional nanostructures.

## 1. Introduction

Drug delivery is always anticipated to be “safe, effective, and convenient” for patients [1,2,3,4,5]. To achieve this goal, drug release profiles must be modified regardless of the drug administration routes [6,7,8,9,10,11]. During the past half a century, both pharmaceutical technologies and excipients broadly expanded to create novel functional materials for realizing a wide variety of modified release profiles of active ingredients, which show potential applications in drug delivery, food engineering, cosmetics, and so on [12,13,14,15,16,17,18,19]. These profiles typically include an immediate release (or pulsatile release or rapid/fast release) [20,21,22,23], sustained release [24,25,26,27,28,29], delayed release [30], biphasic release [31,32,33], and multiple-stage release in terms of the drug release rate. 

Different kinds of modified release profiles have special advantages for therapeutic applications. For example, the immediate release of a drug is essential when rapid action is required, and is welcomed by patients with conditions such as pains or fevers and heart attacks [34,35,36,37,38]. Alternatively, sustained drug release for a long time period is popular with patients for cases where the administration frequency can be reduced for a better quality of life [39,40,41,42,43,44]. Double-stage release, which often includes a fast release followed by an extended release, can combine the advantages of both of these approaches [31,32,33,34,35]. These dosage forms can not only rapidly act to eliminate the symptoms, but can also maintain a relatively constant drug concentration in the blood, which can improve its effectiveness in patients. Thus, it is not unusual that many new methods have been introduced in the preparation of double-stage, release-medicated materials, such as coaxial electrospinning and electrospraying [45,46,47], side-by-side electrospinning [48], and electrospraying [49]. 

Excipients, particularly polymeric excipients, have played a fundamental role in the modification of drug release profiles over the past half a century [50,51,52]. For example, hydrophilic polymers and hydrophobic polymers greatly support the development of fast-release and sustained-release profiles [53,54,55], respectively. Certainly, these polymers need a suitable technique to encapsulate the drug molecules that they contain. Particularly, poorly water-soluble drugs often need to be widely distributed across the polymeric matrices; essentially, these are often composites on the molecular scale [56,57,58,59]. 

Electrospinning, initially a polymer treatment method, has spread its antennas to almost all fields of scientific applications [60,61,62,63,64,65]. For mainstream applications, the functional ingredients are often loaded into the electrospun nanofibers through a co-dissolved solution or melt [66]. The resultant nanofibers are often polymeric composites or a solid dispersion at the molecular scale [67]. However, an increasing number of electrospun hybrids occurred most recently [68]. These electrospun hybrids, on one hand, suggested that the electrospinning method expanded its capability of preparing nanofibers from nano suspensions and emulsions, as well as traditional solutions. On the other hand, the electrospun hybrids may have special advantages over the analogous electrospun nanocomposites. Particularly, electrospun structural hybrids from multiple-fluid electrospinning processes are increasingly studied for a wide variety of scientific applications [69].

Coaxial electrospinning, an updated edition of the single-fluid blending electrospinning, is popular due to its capability of creating core–shell nanostructures [70,71,72,73]. Different from the uni-axial nanofibers, core–shell nanofibers can provide the desired drug-modified release profiles from more adjustable elements, as well as the properties of polymer matrices [74]. These elements not only include the properties of polymeric matrices (the only reliable matrix for homogeneous nanofibers), but also the structural parameters, e.g., the core and sheath polymers or even little molecules, the organizations of the sheath and core materials, the distributions of the functional ingredients, the blank coating, and so on [75]. 

In this study, a modified coaxial electrospinning was implemented to prepare a brand-new nanostructure in which a drug, acyclovir (ACY), was directly coated on a drug–polymer nanocomposite to form a nanohybrid. Polyacrylonitrile (PAN) was selected as the filament-forming polymer of the model, which could be spun into fibers through several types of spinning techniques, including electrospinning, wet spinning, and dry spinning. The prepared ACY-PAN at the ACY nanohybrids were compared with the electrospun ACY-PAN nanocomposites in terms of the morphology and functional performances of modifying drug release profiles. 

ACY is a synthetic, purine, nucleoside analog. It is mainly used for various infections caused by the herpes simplex virus [76]. It can be used for initial or recurrent skin infections, the mucous membrane, external genital infection and herpes simplex virus infection in people with immune deficiency. The incidence and mortality rates of herpes simplex virus drugs are better than those of cytrarabine. This drug can also be used for herpes zoster, Epstein-Barr virus, and immune deficiency complicated with chickenpox. Its oral absorption is poor; about 15% to 30% is absorbed by the gastrointestinal tract. Thus, transdermal administration is an optional mode of administration, and electrospun nanofiber membranes are very convenient for developing transdermal patches [77]. 

## 2. Experimental Section

### 2.1. Materials

PAN (Mw = 80,000) was a gift from Shanghai Jin-shan Petrochemical Co., Ltd. (Shanghai, China). ACY was purchased from Shanghai Huashi pharmacy (Shanghai, China). Red-marker basic fuchsin, acetone, N,N-dimethyl acetamide (DMAc, solvents for preparing working fluids), and phosphate-buffered solution (0.1 M, pH = 7.0) were obtained from Sinopharm Shanghai Chemical Reagent Co., Ltd. (Shanghai, China). Water was double distilled immediately before use.

### 2.2. Preparation of Nanofibers

Two working fluids were prepared for the preparations of nanofibers. The core working fluid was composed of 3% (*w*/*v*) ACY and 15% (*w*/*v*) PAN added into the solvent mixture of DMAc and acetone with a volume ration of 8:2, which was demonstrated to have good electrospinnability by some pre-experiments. The sheath fluid was composed of 10% (*w*/*v*) pure ACY in a mixture of DMAc and acetone (8/2, *v*/*v*). For optimization of the experimental parameters, 5 × 10^−6^ mg/mL of basic fuchsin was added into the core solution. 

Two syringe pumps (KDS100, Cole-Parmer, IL, USA) were utilized to quantitatively drive the core and sheath working fluids separately. A high-voltage generator (ZGF60 kV/2 mA, Wuhan Huate High Voltage Electric Appliance Co., Ltd., Wuhan, Chia) was used to provide the applied high voltage. Cardboard wrapped with aluminium foil was used as a plate fiber collector. The concentric spinneret was homemade. 

The distance between the nozzle of spinneret and the collector was fixed at 20 cm. The applied voltage was fixed at 18 kV. The sheath and core fluid flow rates were adjusted for the preparation of three different nanofiber mats, which are denoted as N1, N2, and N3, respectively (Table 1). All of the resultant nanofiber membranes were stored in a vacuum dryer (DZF6090, Shanghai Precision Instrument Co., Ltd., Shanghai, China) to reach a constant weight. The ambient temperature and relative humidity were (21 ± 5) °C and (46 ± 5)%, respectively. 

### 2.3. Characterizations of Nanofibers

The resultant nanofibers’ morphologies were assessed using a field-emission scanning microscope (FESEM, Hitachi, Tokyo, Japan). A little patch was cut from the electrospun nanofiber membranes and was adhered on the conductive tape on a sample stage. Later, the samples were coated with a thin layer of Au through a gold sputter for 1 min under a nitrogen atmosphere. The inner structures of the prepared nanofibers were evaluated using a field-emission transmission microscope (TEM, JEM2100F, JEOL, Tokyo, Japan). The sampling was prepared by placing a copper-mesh-supported carbon film under the electronic fields for several minutes to collect the nanofibers. 

The physical state of electrospun nanofibers of N1, N2 and N3, and their starting raw materials, ACY and PAN, were assessed using an X-ray (Cu Kα radiation) diffractometer (XRD, Bruker-AXS, Karlsruhu, Germany). The scanning range was from 5° to 60° under a current and voltage of 30 mA and 40 kV, respectively. The compatibility of ACY and PAN within the nanofibers can be evaluated using their Fourier-Transform Infrared (FTIR) spectra. A Perkin-Elmer Spectrum 100 spectrometer (Billerica, MA, USA) was used to carry out the measurements between of 500 cm^−1^ to 4000 cm^−1^. 

### 2.4. Functional Performances 

The ACY concentration (C, μg/mL) in the dissolution media was detected using a UV-2102PC UV-vis spectrophotometer (Unico Instrument Co., Ltd. Shanghai, China). The standard equation between the absorbance (A) and C was C = 0.126A + 0.002 with a correlation coefficient of R = 0.9997, which was built at the maximum absorbance of λmax = 260 nm and within a range from 0.0 to 20.0 μg/mL. 

One concern was the amount of the drug that escaped from the working fluids during the preparation process when a pure drug solution was explored as the sheath solution, i.e., the encapsulation efficiency (EE, %) of the modified coaxial electrospinning. To evaluate the EE values, 50.0 mg electrospun nanofibers were broken and re-dissolved in 20.0 mL DMAc to free all the loaded cargo, and then 1.0 mL of the solution was dripped into 500 mL PBS (0.1 M, pH7.0) to extract the ACY. After detection, the measured content of ACY (*C_M_*) could be calculated from the standard equation, and thus the EE values could be obtained through the following equation:

(1) $$EE=\frac{C_{M}}{C_{T}}\times 100\%$$

where *C_T_* is a theoretical value according to the experimental conditions using the following equation:

(2) $$P_{t}\%=\frac{f_{s}*C_{s-ACY}+f_{c}*C_{c-ACY}}{f_{s}*C_{s-ACY}+f_{c}*(C_{c-ACY}+C_{c-PAN})}$$

where *f* and *C* denote fluid flow rate and solute concentration, respectively; the subscript “*s*” and “*c*” refer to sheath and core, respectively; the compound subscripts “*s-ACY*”, “*c-ACY*” and “*c-PAN*” represent drug concentration in sheath fluid, drug concentration in core fluid, and PAN concentration in core fluid, respectively. 

According to the Chinese Pharmacopoeia (2020 Ed.), a basket method was used to carry out in vitro dissolution tests using a RCZ-8A dissolution apparatus (Tian-Jing University Radio Factory, Tianjin, China). The apparatus contained 7 vessels, in which 800 mL PBS (0.1 M pH7.0) was loaded and maintained at a constant temperature of 37 ± 0.5 °C and a rotation rate of 50 rmp. Six samples were simultaneously placed in six vessels. At the predetermined time points, 5.0 mL of the dissolution medium was withdrawn for analysis; meanwhile, 5.0 mL of the fresh PBS from the seventh vessel was added to maintain a constant volume for dissolution. 

## 3. Results and Discussion

### 3.1. Electrospinning

The simple and straightforward process of electrospinning was modified from different angles. In a single-fluid process, the working fluids must be electrospinnable for the preparation of nanofibers, or the resultant nanofiber would be degraded into electroprayed particles or even wet membranes in the collectors. When two or more simultaneous treatment fluids are used (such as coaxial, side-by-side, tri-axial processes), only one of them must be electrospinnable to support a successful electrospinning process [78,79,80]. In other words, unspinnable fluids can be treated with spinnable fluids simultaneously for the preparation of nanofibers. Thus, these multiple-fluid processes can greatly expand the capability of electrospinning in creating nanofibers, not only from the filament-forming polymers, but also from little molecules [81]. 

**Figure 1:**
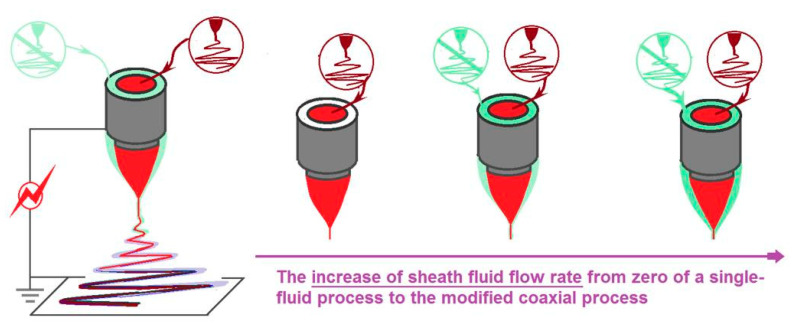
The role of sheath fluid in the modified coaxial electrospinning.

Traditionally, coaxial electrospinning must have an electrospinnable sheath fluid for creating the core–sheath nanostructures. However, Yu et al. deviated from this concept to develop a series of modified, coaxial electrospinning processes [1,2,3,4,5]. Pure solvents, diluted polymer solutions without electrospinnability, phospholipids, and even nanosuspensions, are reported to act as a sheath working fluid to generate novel, functional nanofibers [1,2,3,4,5]. However, no studies were found where pure drug solutions were utilized as sheath-working fluids. Meanwhile, little efforts were paid to the influence of unspinnable sheath fluids on the formations of final nanostructures. Thus, Figure 1 is a diagram that shows the increase in the sheath fluid flow rate from zero (essentially a single-fluid process) to increased values. The as-prepared nanofibers of N1, N2, and N3, were evaluated in order to compare their morphology and drug-modified release profiles. 

**Figure 2:**
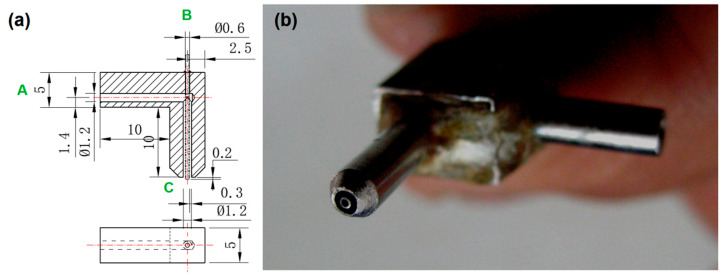
The applied concentric spinneret: (**a**) design of the fabrication details, A and B are the inlets of sheath and core fluids, respectively, and C is their co-exit; (**b**) a digital photo of the concentric spinneret.

To conduct a successful electrospinning process, the apparatus must have a suitable spinneret, which is regarded as the most important and innovative section in an electrospinning system [82]. Figure 2 shows the design (Figure 2a) and a digital photo (Figure 2b) of the spinneret utilized in this study. The key fabrication details in Figure 2a include: (1) a slight projection of 0.2 mm of the inner capillary over the surface of the outer capillary; and (2) the inner capillary with an inner diameter of 0.3 mm, and the outer capillary with a ring thickness of (1.2 − 0.6)/2 = 0.3 mm. The digital image in Figure 2b indicates that the inner capillary and the outer tube have a perfect common axis to form a concentric exit to guide the core and sheath working fluids into the electronic fields. 

**Figure 3:**
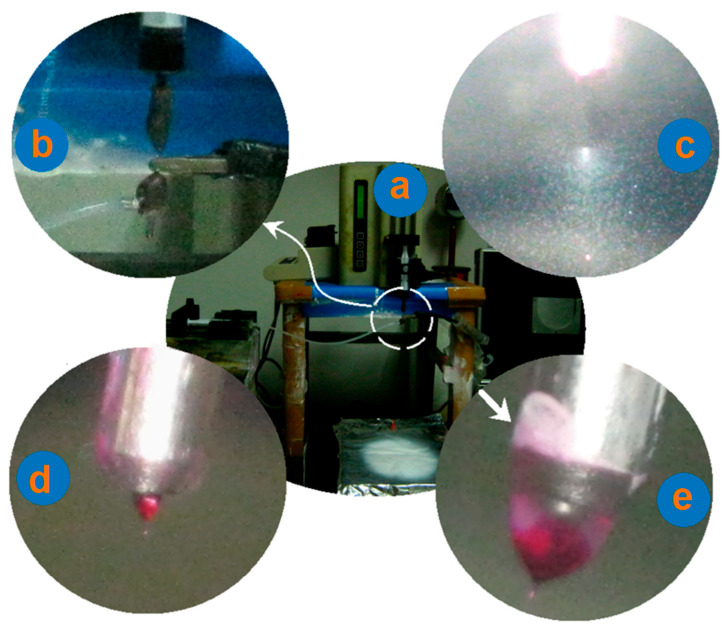
The implementation of modified coaxial electrospinning: (**a**) the organization of the whole electrospinning system; (**b**) a digital photo about the aggregations of working fluids and electrical energy on the spinneret; (**c**) a typical process of electrospraying of the sheath drug solution; (**d**) a Taylor cone that occurred during the single-fluid electrospinning for preparing composite nanofibers of N1; (**e**) a compound Taylor cone taken during the modified coaxial electrospinning for preparing nanohybrids of N2. The arrow indicates drug powders precipitated from the sheath drug solutions.

The arrangement of the electrospinning system is shown in Figure 3a,b. The syringe holding the core fluid can be directly inserted into the spinneret and fixed on a KDS100 pump, whereas another pump can drive the sheath-working fluid to the spinneret through an elastic silicon tube. The collector was placed directly under the concentric spinneret (Figure 3a). An alligator clip was directly connected with the inner capillary to convert the electrostatic energy into the working fluids, as shown in Figure 3b. 

During the electrospinning processes, the key point is the formation of a Taylor cone to initiate an electrospinning process [83,84,85]. Three different working processes were recorded using a digital camera, and are included in Figure 3c–e. Just as anticipated, the sheath drug solution had no electrospinnability. When a voltage of 18 kV was applied solely on the sheath fluid, a typical electrospraying process occurred (Figure 3c). The collector had no precipitated solid particles due to a lower ACY concentration in its DMAc solution. Shown in Figure 3d is a typical Taylor cone during the single-fluid electrospinning process of core drug–polymer, co-dissolved fluid (i.e., the flow rate of sheath ACY solution was zero), through which the electrospun ACY-PAN composite nanofibers of N1 were prepared. Figure 3e is a digital picture of the compound Taylor cone during the modified coaxial electrospinning process for preparing the nanohybrids of N2. The red marker basic fuchsin clearly indicates that the core fluid was surrounded by the sheath solution. Meanwhile, during the working process, the sheath drug solution crawled along the surface of spinneret, which led to the precipitation of some drug particles on the surface of sheath tube, indicated by the arrow in Figure 3e. The preparation of nanofibers of N3 through the modified coaxial electrospinning showed a similar Taylor cone with nanofibers of N2. 

### 3.2. The Morphology and Inner Structure of Electrospun Nano Products 

**Figure 4:**
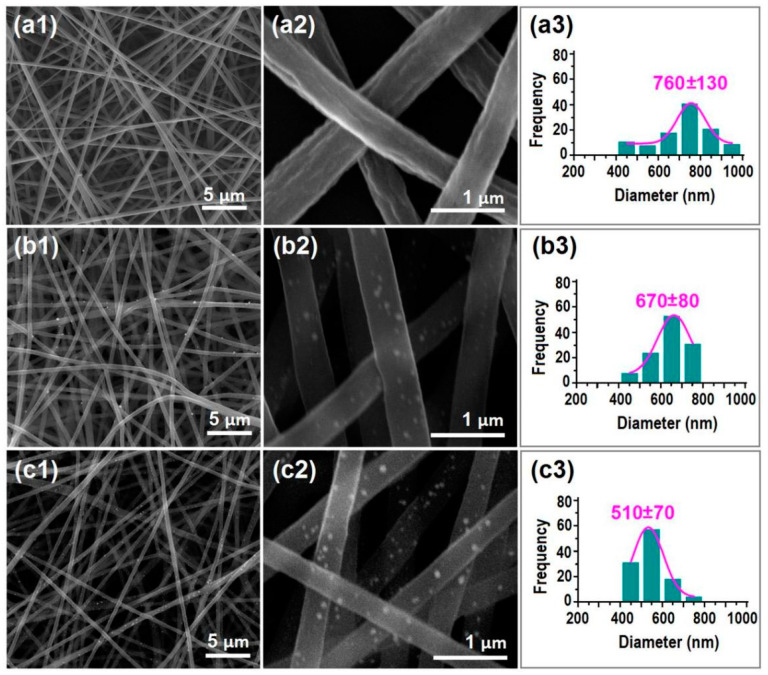
The FESEM assessments of the resultant nanofibers: (**a1**–**a3**) nanofibers of N1, with different magnifications and their size distribution; (**b1**–**b3**) nanofibers of N2, with different magnifications and their size distribution; (**c1**–**c3**) nanofibers of N3, with different magnifications and their size distribution.

The FESEM images of the prepared nanofibers and their size distributions are concluded in Figure 4. All the nanofibers have a fine linear morphology, and the undesired products such as spindles, beads, spindles-on-a-string or beads-on-a-string are seldom observed. This positive result should be attributed to the fine electrospinnability of the core working fluid.

Shown in Figure 4a1,a2 are images of the electrospun composite nanofibers, of N1 under different magnifications. It is clear that the PAN-ACY composite nanofibers’ surface is concave in many places, as shown in Figure 4a2; this is also reported in the literature [86]. These composite nanofibers have an estimated diameter of (760 ± 130) nm. However, when the drug solutions were explored as sheath-working fluids, the prepared nanofibers showed significant differences in the following aspects (Figure 4b1,b2 for nanofibers of N2; Figure 4c1,c2 for nanofibers of N3). (1) Many small nanoparticles were distributed on the surfaces of the nanofibers. These particles should be a result of the recrystallization of ACY molecules on the ACY-PAN surfaces. Additionally, simply because of the presence of these crystalline ACY particles, the resultant nanofibers can be termed as nanohybrids, i.e., a kind of hybrid of polymer–drug composites (core) and a crystalline drug (sheath). Meanwhile, the drug particles did not coat all of the drug–polymer surface, and thus the hybrids are called ACY-PAN at the ACY nanohybrids. (2) The surfaces of electrospun nanofibers of N2 and N3, are smoother than nanofibers of N1; the wrinkles on the surfaces of nanofibers of N1, disappeared from the nanofibers of N2 and N3. 3) With the increase in the sheath fluid flow rate, the nanofibers’ diameter decreased, (670 ± 80) nm (Figure 4b3) and (510 ± 70) nm (Figure 4c3) for nanofibers of N2 and N3, respectively. The sheath solutions were able to prolong the electrical drawing processes of the core ACY-PAN fluids, and thus facilitate the even evaporation of solvents to form smooth nanofibers with a decreased size.

**Figure 5:**
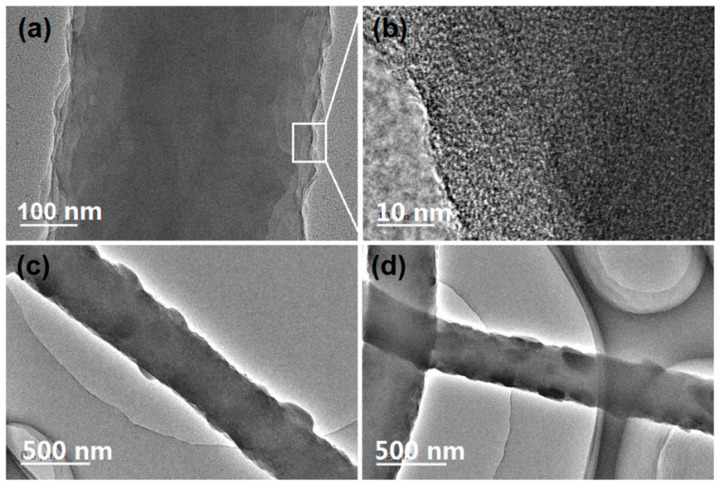
The TEM evaluations of the resultant nanofibers: (**a**,**b**) nanofibers of N1; (**c**) nanofibers of N2; (**d**) nanofibers of N3.

To further explore the inner structures of the electrospun nanofibers from different kinds of working processes, TEM was exploited to evaluate their structures. The results are shown in Figure 5. Figure 5a,b are images of nanofibers of N1, i.e., the electrospun nanocomposites. It is clear that the gray levels are homogeneous and gradually decrease from the fiber centers to the boundaries due to fiber thicknesses. The enlarged images in Figure 5b indicate that there are no separated ACY particles from the ACY-PAN composites during the electrospinning and later storage processes. In sharp contrast, both nanofibers of N2 and N3, have some particles on their surface, which are shown in Figure 5c,d, respectively. In contrast, the recrystallized particles on nanofibers of N2 are larger than those on nanofibers of N3. The smaller sheath flow rate may result in a fast recrystallization of ACY molecules and the formation of particles with a large size.

### 3.3. The Physical State of Components and Their Compatibility 

**Figure 6:**
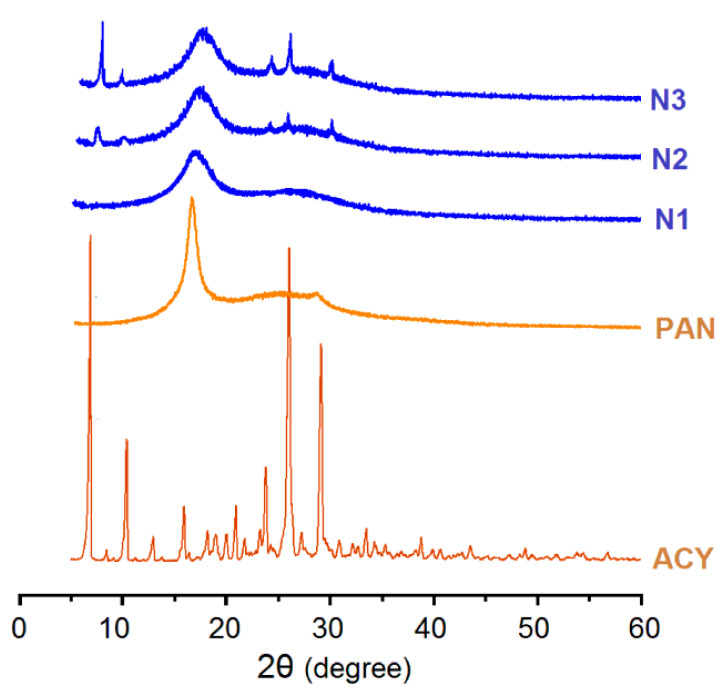
The XRD patterns of the drug, ACY; polymer, PAN; their composites of N1; and their nanohybrids of N2 and N3.

To further disclose the physical state of ACY presented in the electrospun composites of N1, and the electrospun N2 and N3 hybrids of acyclovir-polyacrylonitrile at acyclovir, XRD patterns were achieved, and the results are shown in Figure 6. The raw ACY powders, with a white color and smaller than 20 μm, have many sharp peaks in their patterns, initially suggesting a crystalline material. PAN powders have a sharp peak, suggesting that there are some orientations of PAN molecules in them. However, in the electrospun nanocomposites of ACY-PAN, all the ACY peaks in the patterns of its powders disappeared, and the sharp peaks in the patterns of PAN powders were greatly reduced. These phenomena suggest that ACY and PAN molecules formed molecule–molecule composites, and the original crystal nucleus and orientations were replaced by ACY-PAN amorphous composites. In contrast, some peaks can be still detected from the electrospun nanohybrids of N2 and N3. These peaks suggest that there are ACY crystalline particles presented on the electrospun nanofibers of N2 and N3, demonstrating that they are electrospun structural hybrids of ACY-PAN at ACY. 

**Figure 7:**
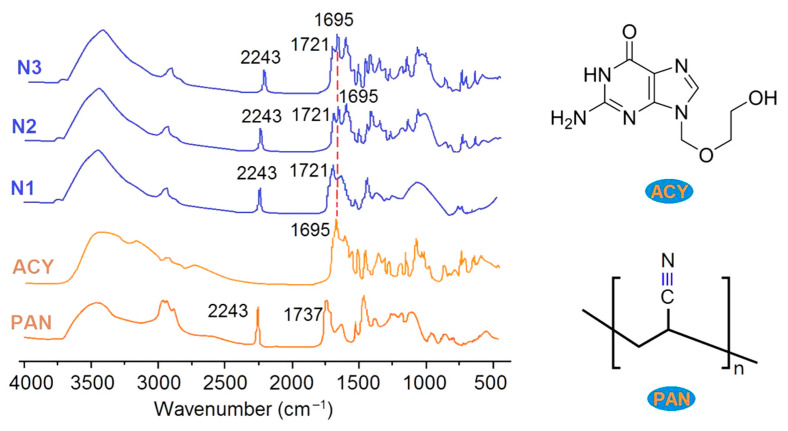
The FTIR spectra of the drug, ACY; polymer, PAN; their composites of N1; and their nanohybrids of N2 and N3, as well as the molecular formula of ACY and PAN.

The FTIR spectra of the drug, ACY; polymer, PAN; their composites of N1; and their nanohybrids of N2 and N3, are shown in Figure 7. The wavenumber of 2243 cm^−1^ indicates the presence of –C≡N groups, which is a characteristically functional group of PAN molecules. In PAN powders, there are often residues left during the synthesis processes, in which there are some –C=O groups, and thus there is a sharp peak at 1737 cm^−1^. In the FTIR spectra of ACY powders, the sharp peak at 1695 cm^−1^ indicates the –C=O group in the ACY molecules (Figure 7), and there are many sharp peaks in its fingerprint region. In comparison, there are three changes in the spectra of electrospun nanocomposites of ACY and PAN: (1) the sharp peaks in the finger region of ACY reduced greatly and even disappeared; (2) the sharp peak at 1695 cm^−1^ is not clear; (3) there was a red shift from 1737 cm^−1^ to 1721 cm^−1^ for the PAN’s residues. These changes verified that PAN and ACY formed molecular composites through secondary interactions such as hydrogen bonding, electrostatic interactions and hydrophobic interactions, which could also be deduced from molecular structures (Figure 7). Compared with the spectra of electrospun nanocomposites of N1, the main differences present in the spectra of electrospun nanohybrids of N2 and N3, include these phenomena. One phenomenon is the height increase in the sharp peak at 1695 cm^−1^, and the other is the peaks in the finger region. These differences demonstrate that the electrospun nanofibers of N2 and N3, are a hybrid of crystalline ACY powders and amorphous ACY-PAN composites. The more drug that is loaded on the sheaths of amorphous PAN-ACY nanofibers, the stronger the peaks at 1695 cm^−1^, and the singer region shows a clear result from the comparison of nanohybrids of N2 and N3. 

### 3.4. The Functional Performances of Modifying the Drug Release Profiles 

The measured drug loadings in electrospun nanofibers of N1, N2, and N3 are 17.1 ± 0.4%, 23.9 ± 0.4%, and 30.2 ± 0.5%, respectively. The calculated drug loadings according to the experimental conditions are 16.7%, 24.1%, and 29.9%, respectively. Thus, the drug encapsulation efficiency (EE, %) are 102.4%, 99.2%, and 101.0%, respectively. The results suggest that there is no drug loss during the electrospinning processes, regardless of the numbers of working fluids and implementation formats. The electrospinning process is essentially a physical drying process and the drug, ACY, has no volatile and sublimable properties, and thus it is just as anticipated that all the drug dissolved in the working fluids is encapsulated in the resultant nanofibers.

**Figure 8:**
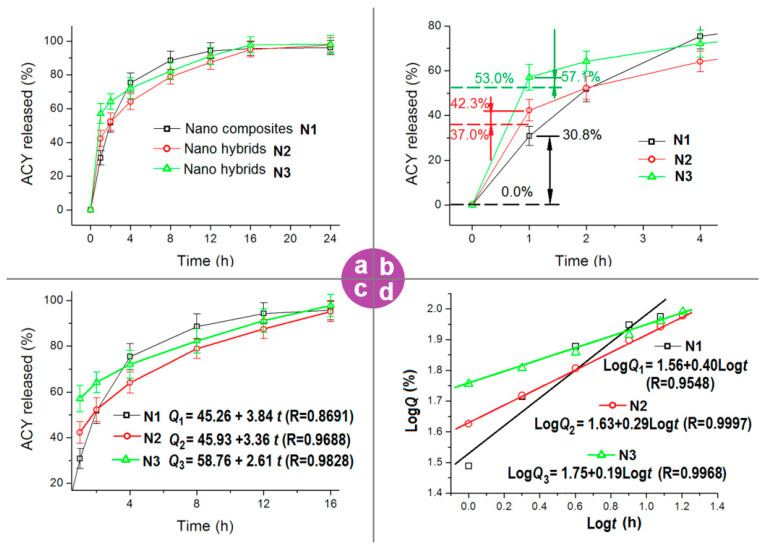
The modified release profiles of ACY by the composites of N1, the nanohybrids of N2 and N3, and the related analyses: (**a**) release profiles; (**b**) release profiles at the initial 2 h; (**c**) regressed results according to the zero-order kinetic equation; (**d**) regressed results according to the Peppas equations.

The ACY release profiles from the three kinds of electrospun nanofibers are included in Figure 8a. All the nanofibers can provide an extended release time period of over 16 h. However, significant differences can be discerned after further treating these data. An enlarged image of the first 2 h of initial release profiles is shown in Figure 8b. In the first hour, an accumulative release of 30.8%, 42.3%, and 57.1% was measured for nanofibers of N1, N2 and N3, respectively. 

The 30.8% release at the first hour from the electrospun ACY-PAN nanocomposites represents a typical initial burst release, which is a negative phenomenon in the drug-sustained release profile. This is because the initial burst release is uncontrollable. The reasons for this are attributed to a homogeneous distribution of the drug all over the polymeric matrix, a large surface area of the nanofibers, and either a short distance or no distance for the drug molecules solidified on the surface of nanofibers to dissolve into the bulk dissolution media. As for the electrospun nanohybrids of N2 and N3, the theoretical cargoes of drug on the sheath can be calculated according to the experimental conditions and Equation (2), i.e., 37.0% and 53.0%, respectively. The detected amounts released are 42.3% and 57.1%, respectively. These data mean that (42.3% − 37.0%) = 5.3% and (57.1% − 53.0%) = 4.1% deviate from the designed values. These smaller deviations suggest that the electrospun nanohybrids are preferable for developing precision medicine and personalized medical products.

To disclose the mechanisms from which the electrospun nanofibers modified the drug release behaviors, both zero-order and Peppas equations are explored to treat the in vitro dissolution data. The results achieved from the zero-order kinetic equation are shown in Figure 8c. For nanofibers of N1, N2, and N3 (from 1 h to 16 h), the equations are Q_1_ = 45.26 + 3.84*t* (R = 0.8691), Q_2_ = 45.93 + 3.36*t* (R = 0.9688) and Q_3_ = 58.76 + 2.61*t* (R = 0.9828), respectively (where Q and t denote the accumulative release amount as a percentage and the release time period in hours, respectively). From a comparison of their correlation coefficients, it is clear that the electrospun nanohybrids of N2 and N3 exhibited a better performance than the nanocomposites of N1 in modifying the ACY molecules release in a zero-order format after the initial first hour release. 

The regressed Peppas equations [87] of the electrospun nanofibers are included in Figure 8d. For nanofibers of N1, N2 and N3 (from 1 h to 16 h), the equations are LogQ_1_ = 1.56 + 0.40*t* (R = 0.9548), LogQ_2_ = 1.63 + 0.29*t* (R = 0.9997) and LogQ_3_ = 1.75 + 0.19*t* (R = 0.9968), respectively. The excipients of electrospun nanofibers of N1, N2 and N3 are 0.40, 0.29, and 0.19 (all smaller than the critical value of 0.45, which is often utilized to judge an erosion or a diffusion mechanism), respectively, suggesting that the ACY molecules freed to the dissolution media from the PAN matrix were solely controlled by the Fickan diffusion mechanism. This was expected because the PAN molecules neither dissolved into water nor degraded over time in the dissolution media. Additionally, ACY is a typical poorly water-soluble drug. Compared with traditional strategies for resolving the important issue of the dissolution and delivery of poorly water-soluble drugs, such as drug nanocrystals, drug–polymer solid dispersions, homogeneous drug composites, and drug–polymer conjugates [88,89], the reported nanohybrids demonstrate a new way of combining drugs and polymers, and, correspondingly, provide an alternative way of modifying the release profiles of poorly water-soluble drugs. 

## 4. Conclusions

In the present study, a modified coaxial electrospinning was carried out to prepare two sorts of ACY-PAN at the ACY nanohybrids. An electrospun ACY-PAN nanocomposite was prepared using a single-fluid blending process for comparison. FESEM measurements demonstrated that all of the nanohybrids and nanocomposites presented in a linear morphology without beads or spindles on the string. TEM observations showed that the nanohybrids had no wrinkles and occurred on the composite nanofibers N1, but there are many separated ACY particles. XRD results verified that these particles were crystalline nanoparticles of ACY, and FTIR spectra verified that the drug ACY and the polymer PAN have a good compatibility. In vitro dissolution tests disclosed that the electrospun ACY-PAN at the ACY nanohybrids of N2 and N3 from the modified coaxial showed a better performance in modifying the drug release profiles, compared to the electrospun ACY-PAN nanocomposites of N1, which mainly presented in the following three aspects. One aspect is that the initial release amount is more accurately and intentionally controlled. The second is that the sustained release in the second step is nearer to the zero-order kinetic equation. Last but not least, the in vitro dissolution test results of the nanohybrids of N2 and N3, demonstrated that the release amount from the first and second stages can be easily allocated through the sheath fluid flow rate. The present protocols should pave a new way for developing new types of functional nanomaterials. 

## Figures and Tables

**Table 1:** Parameters for preparing the hybrid medicated nanofibers.

No.	Process	High Voltage (kV)	Flow Rate (mL/h)		Morpho-Logy ^c^	Drug ^d^ (wt%)	Ratio ^d^
			Sheath ^a^	Core ^b^			Stage I/II
N1	Uniaxial	18	--	2.0	Linear	16.7%	0:100
N2	Coaxial	18	0.3	1.7	Linear	24.1%	0.37:0.63
N3	Coaxial	18	0.5	1.5	Linear	29.9%	0.53:0.47

^a^ A ratio of 3% (*w*/*v*) ACY and 15% (*w*/*v*) PAN were dissolved into the solvent mixture for preparing the core spinnable fluid. ^b^ A ratio of 10% (*w*/*v*) ACY was dissolved into the solvent mixture for the preparation of the sheath unspinnable fluid. ^c^ “Linear” means that the prepared nanofibers presented in a straight linear manner with few beads or spindles on them. ^d^ Theoretical calculation values according to the experimental conditions. Stage I and II refer to the initial release stage and the later sustained release stage, respectively.

## Data Availability

The data supporting the fifindings of this manuscript are available from the corresponding authors upon reasonable request.
