# Correction: Lv et al. Electrospun Structural Hybrids of Acyclovir-Polyacrylonitrile at Acyclovir for Modifying Drug Release. *Polymers* 2021, *13*, 4286

**DOI:** 10.3390/polym18010035

**Published:** 2025-12-23

**Authors:** He Lv, Shiri Guo, Gaoyi Zhang, Wanli He, Yonghui Wu, Deng-Guang Yu

**Affiliations:** 1 School of Materials and Chemistry, University of Shanghai for Science and Technology, Shanghai 200093, China; 2 School of Optical-Electrical and Computer Engineering, University of Shanghai for Science and Technology, Shanghai 200093, China; 3 The Department of Mechanical Engineering, Guangxi Technological College of Machinery and Electricity, Nanning 530007, China; 4 Shanghai Engineering Technology Research Center for High-Performance Medical Device Materials, Shanghai 200093, China

In the original publication [1], there were mistakes in Figure 6, i.e., there was no y-axis for the XRD patterns and the noises of the N2 and N3 patterns were highly similar. The reasons for the previous failure to include a y-axis include, but are not be limited to, the following: (1) the XRD here is a qualitative analysis; (2) the only purpose of the XRD patterns is to disclose the physical state of drug molecules in the electrospun nanofibers based on the presence (or not) of sharp Bragg peaks [2,3,4,5,6,7]; (3) sampling is not identical for all the samples; (4) the y-axis’ physical quantity has a unit-less intensity, and its values and the related noises have no scientific meanings, suggest nothing, form no judgments, and have no influence on the conclusions; (5) it is often very hard to include all the XRD patterns in one figure using the same scale range, or some XRD patterns have become straight smooth lines; (6) numerous publications have no y-axes for their XRD patterns [8,9,10]. However, the presence of a y-axis may make understanding the XRD patterns easier for the readers, and moreover, there should be two coordinate systems in one figure. Thus, we have re-prepared the samples and retested their XRD patterns, which are corrected in Figure 6, as shown below.

The authors state that the scientific conclusions are unaffected. The corrections were approved by the Academic Editor. The original publication has also been updated.

**Figure 6:**
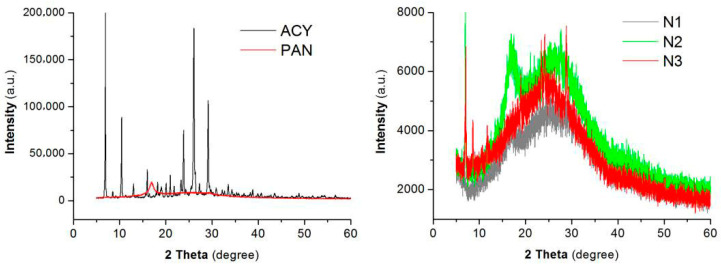
The XRD patterns of the drug, ACY; polymer, PAN; their composites of N1; and their nanohybrids of N2 and N3.
